# Utilizing birch leaf extract in pickling liquid as a sustainable source of corrosion inhibitor for pipeline steel

**DOI:** 10.1038/s41598-022-23037-8

**Published:** 2022-11-11

**Authors:** Q. Mohsen, M. A. Deyab

**Affiliations:** 1 Department of Chemistry, College of Sciences, Taif University, Taif, Saudi Arabia; 2 Egyptian Petroleum Research Institute (EPRI), Nasr City, Cairo, Egypt

**Keywords:** Electrochemistry, Green chemistry

## Abstract

This study set out to determine the effectiveness of birch leaves extract (BLE) as a corrosion inhibitor against X52 pipeline steel in the pickling solution. Chemical and electrochemical techniques, as well as scanning electron microscope (SEM), Fourier-transform infrared (FT-IR), and adsorption isotherms were used in the research. Various triterpenoids, including betulin, betulinic acid, oleanolic acid, sitosterol, and kaempferol, are unquestionably involved in the corrosion inhibition mechanism, according to the high-performance-liquid-chromatography (HPLC) analysis. The 95% efficiency of the produced BLE extract (at optimum concentration 400 mg L^−1^) significantly reduced the corrosion rate of X52 pipeline steel in the pickling solution. The adsorption of BLE extract molecules on the X52-steel surface was demonstrated by SEM and FT-IR analysis. The adsorption activity follows the Langmuir adsorption theory.

## Introduction

Steel is a popular choice for oil and gas pipeline fabrication because of its excellent durability and mechanical power^1^. Petroleum internal pipeline corrosion is caused by a variety of reasons including O_2_ concentration, chlorine ions, sulphur dioxide ions, and hydrogen sulphide^2,3^. Corrosion defects have a variety of risks, including fatal accidents, environmental negative effects, pollutants, and shutdowns. To eliminate oxides from the pipelines surface, acid solutions are utilized. Pickling chemicals like as hydrochloric acid are primarily used to clean petroleum pipelines of oxide layer^4^. When compared to sulfuric acid, the benefits of utilizing HCl include shorter pickling times, less temperatures, and improved surface smoothness with lower pickling degradation. The corrosive nature of the pickling chemicals causes the pipelines to deteriorate in general^5^. Insight into the mechanisms of corrosion and utilizing corrosion inhibitors can dramatically lessen the negative impacts of pickling chemicals^6–8^. Organic chemicals, which contain hetero-atoms including S, N, and O, are effective inhibitors^9–11^.

Toxic chemicals' use as corrosion inhibitors has recently been restricted due to environmental concerns. As a result, natural inhibitors have re-emerged as significant due to their ecologically benign character, ease of production, and renewable origins^12–15^. According to a review of the literature, there are several attempts at this issue. Su et al.^16^ extracted the active ingredients from gingko leaves using an ultrasonic device. The anti-corrosion effectiveness reached 84.7%. Sun et al.^17^ created corrosion inhibitors using pomelo peel. They investigated the corrosion of N80 inside a 3.5 wt% saline solution saturated with CO_2_. The investigation demonstrated that physical adsorption was indeed the primary adsorption tendency, with maximum effectiveness of about 87%. Arash et al.^18^ investigated the corrosion protection of lemon verbena leaves in a mixed HCl and H_2_S solution. There was evidence of physical adsorption. The greatest efficiency was 95.61% at the concentration reached 2.7 g L^−1^. Hassannejad et al.^19^ examined the corrosion control of sunflower seed husk extracts in hydrogen chloride for carbon steel. This extract appears to contain the O, N, and aromatic ring groups. After the extract was added, the inhibition rate increased to 98.5%. As a result, the strategic goal of this study was to evaluate the birch leaves extract (BLE) to reduce the corrosion of X52 pipeline steel (X52-steel) in pickling solution (1.0 M HCl).

Birch leaves are found in northern temperate and boreal climes most of the time. They are modest in cost and contain high amounts of organic chemicals. It is also less expensive than other plants. Furthermore, no previous research has been conducted on the use of Birch leaves as a corrosion inhibitor in pickling solutions.

## Experimental details

### Materials

The chemical content of the X52 pipeline steel (X52-steel) was as follows (wt. %): C(0.24%), P(0.023%), S(0.024%); Mn(1.2%), Ni (0.240%), Cr(0.34%), Cu (0.40%), Fe(balance%). It comes from an Egyptian petroleum company. The X52-steel surface was physically scraped away with increasing grit SiC sheets before each experiment, then ultrasonically washed in ethanol, fully washed with water, and air dried. 1 M HCl (AR grade 37%) solution was used to make the pickling solution**.**

Birch leaves powder was delivered by an Egyptian herbal family group. For 5 h, 25 g of birch leaves powder was refluxed in 100 ml of the stock mixture (70% C_2_H_5_OH + 10% ethyl acetate + 20% distilled H_2_O) at 358 K. Following that, the refluxed mixture was filtered using a separator funnel. The mixture was then concentrated in a rotary vacuum evaporator and dried in a pressure dry oven at 333 K for 24 h. Lastly, the precipitate was left to dry in a desiccator. Liquid chromatography (shimadzu co. japan) and FTIR (Perkinelme co.) were used to determine the major components of BLE extract. The BLE extract is completely soluble in pickling solution.

### Corrosion rate tests

Both chemical and electrochemistry approaches were used to assess the corrosion rates of the X52-steel in testing solutions.

Weight loss assessments were taken by weighing the dry X52-steel (dimension 3.0 × 3.0 × 0.1 cm) on an electronic balance and immersing it in testing solutions for 5.0 h. The weight loss was estimated by subtracting the weight of the sample before and upon immersion in the investigated solutions.

The electrochemical investigations were conducted using a Gamry 3000 electrochemical station outfitted with a standard three-electrode setup. The working electrode was an X52-steel piece (electrode area = 0.445 cm^−2^), the counter electrode was a platinum plate, and the reference electrode was a saturated calomel electrode (SCE). The tests were conducted out in a temperature-controlled water bathtub. On the *E*_OCP_, electrochemical impedance spectroscopy (EIS) was used. A 10 mV peak-to-peak sinusoidal wave in the frequency region of 100,000–0.01 Hz was used. Gamry's software was used to fit and analyze the EIS data.

### Surface characterization

SEM (ZEISS/EVO) equipped with an EDX analyzer was used to examine the morphologies of X52-steel surfaces in pickling solutions.

## Results and discussion

### Major components of BLE extract

**Figure 1:**
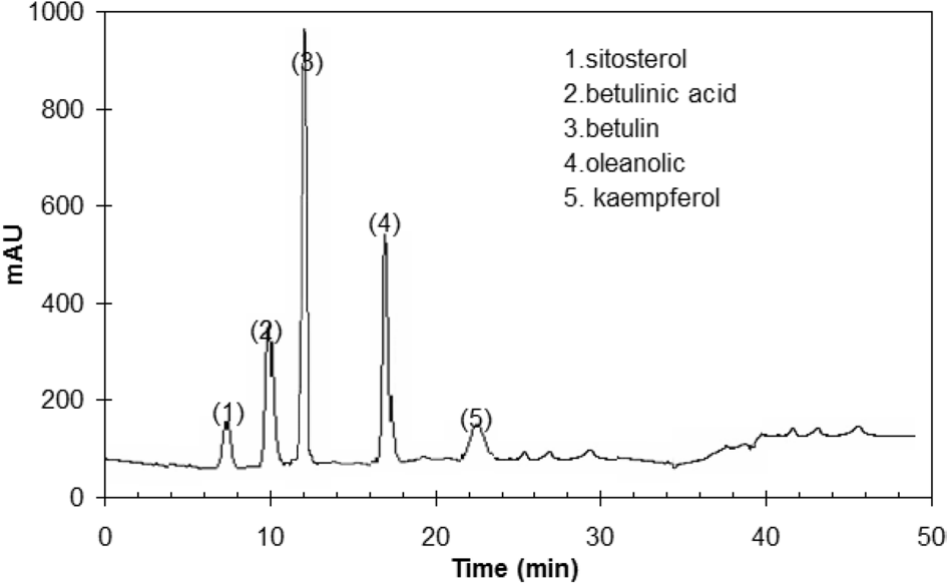
HPLC of BLE extract.

**Figure 2:**
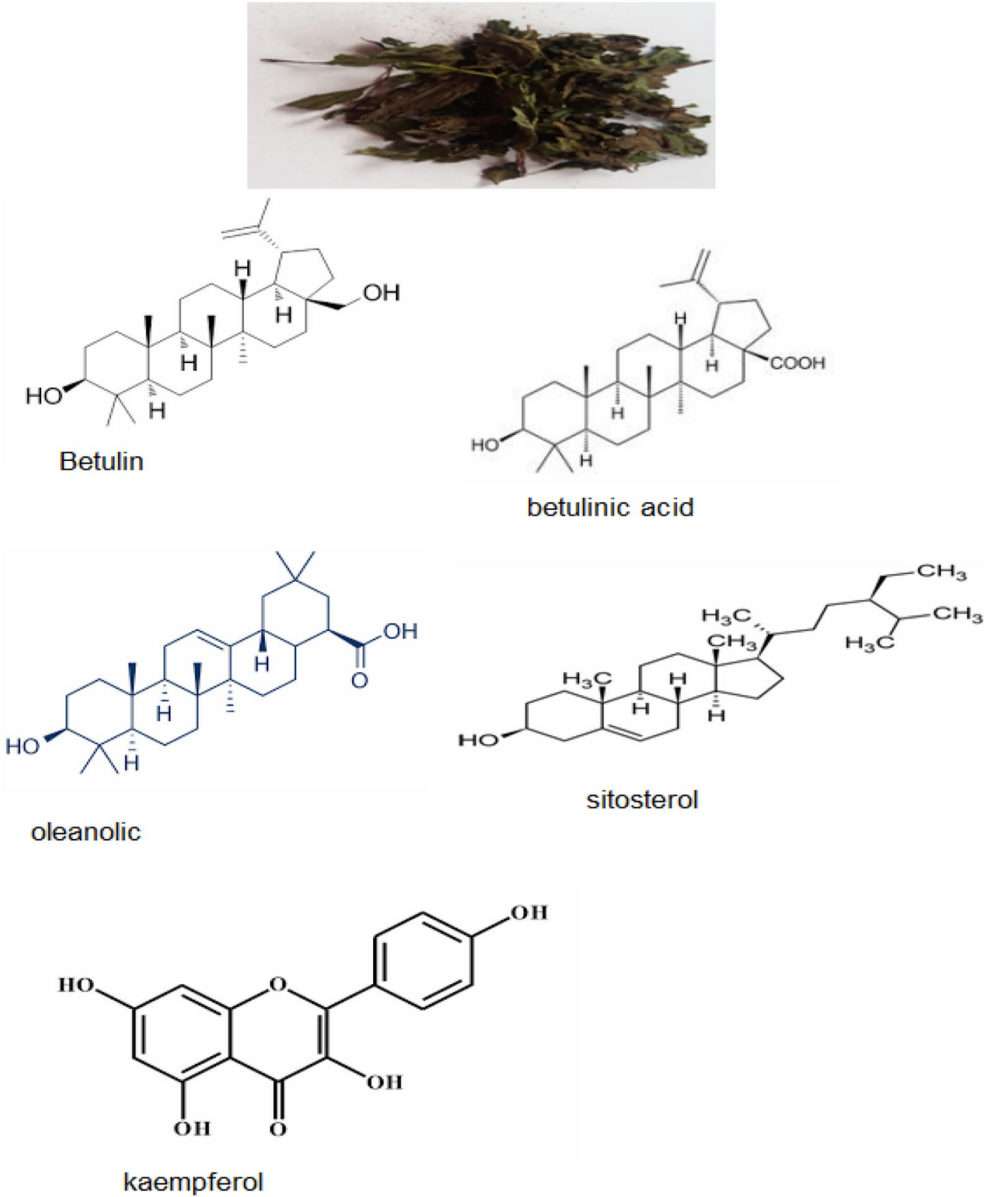
Chemical structures of the key components of BLE extract.

The HPLC (Fig. 1) showed that the major components of BLE extract are (77.9%) triterpenoids (i.e. betulin, betulinic acid, oleanolic) and (2.3%) sitosterol, and (1.3%) kaempferol. Figure 2 depicts the chemical structures of the key components of BLE extract.

**Figure 3:**
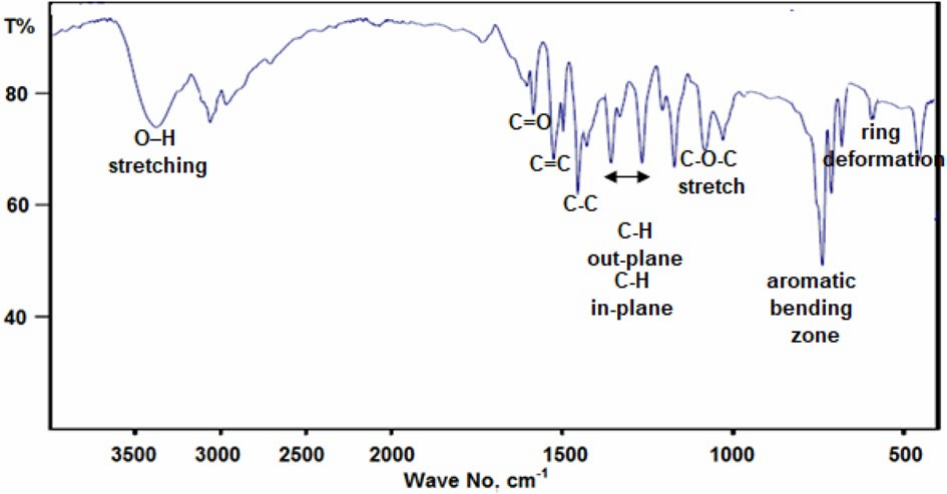
FT-IR spectrum of BLE extract.

The FT-IR spectrum of BLE extract is presented in Fig. 3. A broad band at 3375 cm^−1^ assigned to hydrogen-bonded O–H stretching, the bands at 1684, 1528, 1453, 1267, 1250, 1170, 730, and 686 cm^−1^ are due to C=O, C=C, C–C, C–H in-plane bending, C–H out plane bending, C–O–C stretch, aromatic bending zone, and ring deformation out of the plane, respectively.

### Weight loss study

Weight loss studies were done to examine the influence of BLE extract on X52-steel corrosion in pickling solution (1.0 M HCl). Different doses of the BLE extract were injected into the experimental solution for an estimation of the inhibition activity, and the test was conducted at temperatures of 298 K. Table 1 displays the results. The next formulas are used to compute the experiment variables of corrosion rate (v), inhibition efficiency (*η*_W_%), and surface coverage (*θ*)^20–22^.

1 $$\nu = \frac{{\text{W}}}{{{\text{St}}}}$$

2 $$\theta = \frac{{\nu_{0} - \nu }}{{\nu_{0} }}$$

3 $$\eta_{{\text{w}}} \% = \frac{{\nu_{0} - \nu }}{{\nu_{0} }} \times 100$$

**Table 1:** Percentage corrosion inhibition efficiency of varied concentrations of BLE extract for X52-steel in 1.0 M HCl at 298 K calculated by weight loss method.

BLE (mg L^−1^)	ν (μg cm^−2^ min^−1^)	*θ*	*η*_W_%
Blank	20.23 ± 0.43		–
25	15.80 ± 0.40	0.217	21. 7
50	12.77 ± 0.32	0.368	36.8
100	5.81 ± 0.29	0.712	71.2
200	2.16 ± 0.30	0.893	89.3
300	1.13 ± 0.22	0.944	94.4
400	1.09 ± 0.13	0.946	94.6

where W = weight loss, S = total surface area of X52-steel, t = immersion time, *v*_*0*_ and *v* are the corrosion rate without and with BLE extract, respectively.

According to Table 1, raising the concentration of BLE extract decreases the corrosion rate, increases the inhibitor effectiveness, and increases the coverage, indicating that an increase in the BLE extract concentration leads to a higher degree of adsorption^23^. The inhibitor power of BLE extract increases to 94.4% when the concentration approaches 300 mg L^−1^, confirming that BLE extract is an efficient inhibitor of X52-steel corrosion in the pickling solution. When the concentration of BLE extract approaches 400 mg L^−1^, there is no appreciable decrease in corrosion rate.

### Polarization study

In pickling solution (1.0 M HCl), X52 steel corrosion is essentially a combined electrochemical reaction between anodic (Eq. 4) and cathodic (Eq. 5) reactions.

4 $${\text{Fe }} \to {\text{ Fe}}^{2 + }_{{({\text{aq}}{.})}} + \, 2{\text{e}}$$

5 $${\text{H}}^{2 + }_{{{\text{(aq}}{.)}}} + \, 2{\text{e }} \to {\text{H}}_{2} \left( {\text{g}} \right)$$

**Figure 4:**
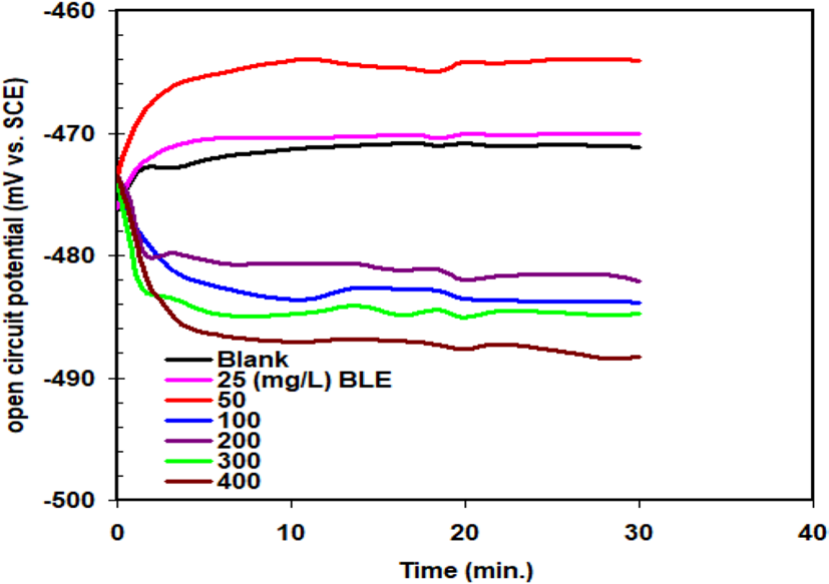
OCP–time curves for X52-steel corrosion in pickling solution (1.0 M HCl) with and without varying concentrations of BLE extract at 298 K.

The open circuit potential (OCP) vs. time for different BLE extract concentrations is depicted in Fig. 4. They have been compared to a blank curve produced under comparable environments. The OCP is clearly balanced after 30 min, as shown in Fig. 4, and the process also seems to have achieved steady state. The change of the curve towards the negative potential area caused by the high concentrations of BLE extract (100 mg L^−1^) in the solution showed that BLE extract has an impact on cathodic reaction.

**Figure 5:**
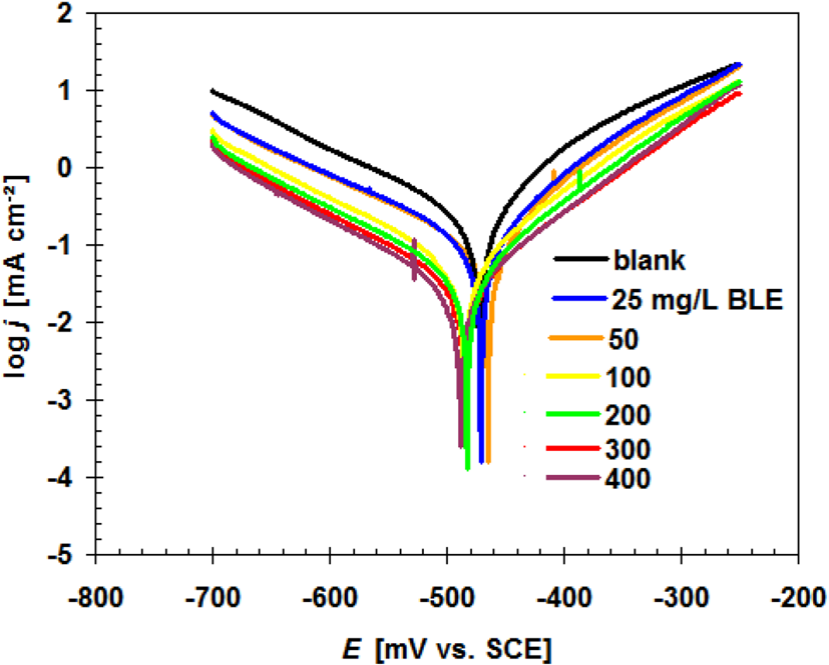
Polarization curves for X52-steel corrosion in pickling solution (1.0 M HCl) with and without varying concentrations of BLE extract at 298 K.

The polarization curves for X52-steel corrosion in pickling solution (1.0 M HCl) with and without varying concentrations of BLE extract at 298 K and a scan rate of 0.125 mV s^−1^ are seen in Fig. 5. Table 2 shows the electrochemical variables of corrosion potential (*E*_corr_), corrosion current density (*j*_corr_), Tafel slopes (*β*a, *β*_c_), assessed using Tafel extrapolation, as well as the inhibition efficiency (*η*_j_%) calculation by the following formula^24^.

6 $$\eta_{{\text{j}}} \% = \frac{{j_{{{\text{corr}}(0)}} - j_{{{\text{corr}}}} }}{{j_{{{\text{corr}}(0)}} }} \times 100$$

**Table 2:** Percentage corrosion inhibition efficiency and polarization parameters of varied concentrations of BLE extract for X52-steel in 1.0 M HCl at 298 K calculated by polarization method.

BLE (mg L^−1^)	*E*_corr._ mV (SCE)	*j*_corr._ (μA cm^−2^)	*β*_a_ (mV dec^−1^)	− *β*_c_ (mV dec^−1^)	*η*_j_%
Blank	− 471	629.3	72.4	165.6	–
25	− 470	422.2	84.3	173.7	32.9
50	− 464	368.1	87.8	134.7	41.5
100	− 484	145.9	92.8	188.7	76.8
200	− 482	61.6	98.8	182.5	90.2
300	− 485	32.0	101.3	178.9	94.9
400	− 488	30.6	105.3	189.4	95.1

where the *j*_corr(0)_ is the corrosion current density without BLE extract.

Table 2 clearly shows that as the content of BLE extract grew, the inhibition efficiency (*η*_j_%) improved while the value of corrosion current density (*j*_corr_) reduced rapidly, particularly at higher concentrations. Table 2 illustrates that the change values of *E*_corr_ versus blank solution were less than 85 mV, revealing that the BLE extract becomes a mixed-type inhibitor^25,26^. The fact that the injection of BLE extract has no strong influence on the Tafel slopes (a, c) implies that the BLE extract's inhibitor has no direct impact on the corrosion mechanism^27,28^.

Once the BLE extract concentration was raised to 400 mg L^−1^, the *η*_j_% value attained 95.1%, indicating that the BLE extract has a strong anti-corrosion ability.

### EIS study

**Figure 6:**
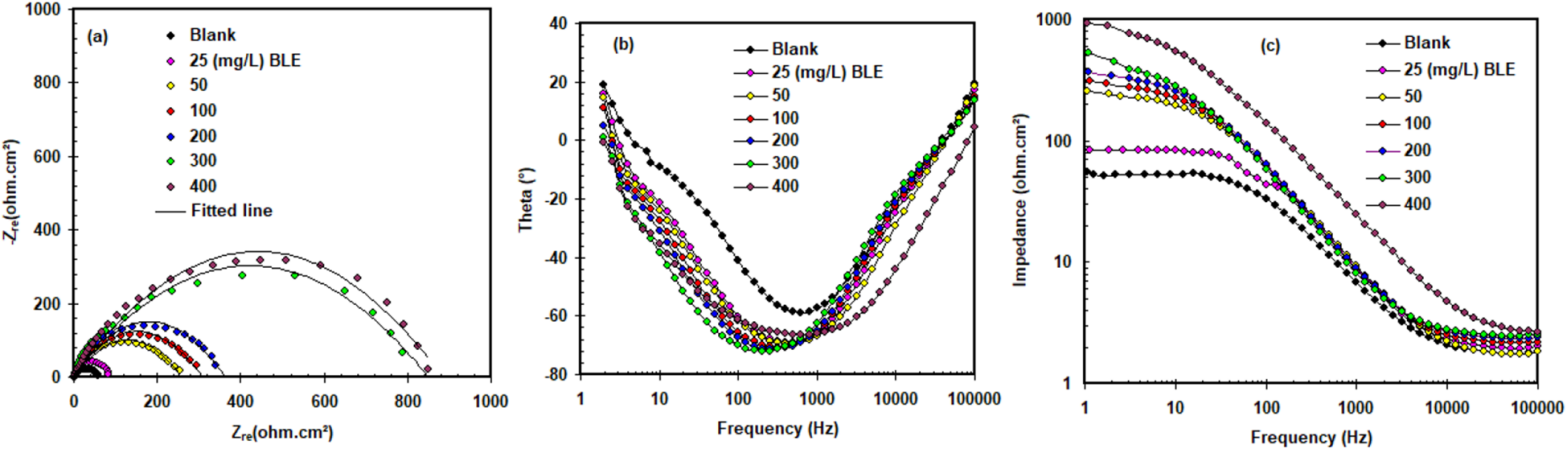
Impedance spectra includes (**a**) Nyquist, (**b**) Bode-phase angle and (**c**) Bode-module plots for X52-steel corrosion in pickling solution (1.0 M HCl) with and without varying concentrations of BLE extract at 298 K.

To verify the polarization results, impedance tests for X52-steel corrosion in pickling solution (1.0 M HCl) with and without BLE extract were investigated (see Fig. 6). The Nyquist plot (Fig. 6a) reveals a depressing capacitive circle, and as the BLE extract concentration increases, the size of the capacitive circle expands, implying that the BLE extract significantly restricts charge transfer between the pickling solution and X52-steel^29^. Furthermore, the fact that the pattern of the impedance spectra does not vary with the presence of BLE extract implies that the addition of the BLE extract does not affect the corrosion mechanism^30–32^. Figure 6b depicts phase angle plots for X52-steel corrosion in pickling solution (1.0 M HCl) with various concentrations of BLE extract. The phase angle plot demonstrates a growing trend as the concentration of BLE extract increases. The results revealed that increasing the BLE extract content up to 400 mg L^−1^ strengthened the protective barrier qualities of the BLE extract layer^33^. The appearance of a high and low-frequency plateau can be seen in the Bode-modulus graphs (Fig. 6c). The radii of the capacitive semicircles and the values of the low-frequency plateau are both consistent.

**Figure 7:**
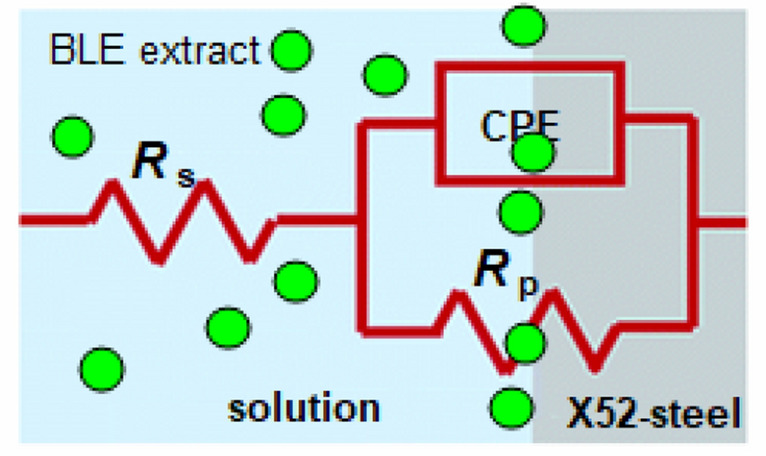
The equivalent circuit for fitting of the impedance data.

The equivalent circuit type with and without BLE extract can be seen in Fig. 7 by fitting the results were significant, which includes the solution resistance (*R*_S_), and polarization resistance (*R*_p_). Furthermore, CPE is a constant phase that represents the double layer capacitance (*C*_dl_) at the contact of the X52-steel and pickling solution. To produce a more precise fit of the experimental given data, CPE was incorporated^34^. Equation (7) provided estimates for the *C*_dl_ values.

7 $$C_{{{\text{dl}}}} = \, \left[ {{\text{Y}}^{0} R_{{\text{p}}}^{{({1} - {\text{n}})}} } \right]^{{{1}/{\text{n}}}}$$

where Y0 = admittance.

Table 3 presents the EIS parameters of various concentrations of BLE for X52-steel in 1.0 M HCl at 298 K, and the inhibitory efficacy (*η*_R_%) was computed using the Eq. (8).

8 $$\eta_{{\text{R}}} \% \, = \, \left[ {\left( {R_{{\text{p}}} - R_{{{\text{p}}(0)}} } \right)/\left( {R_{{\text{p}}} } \right)} \right] \, \times { 1}00$$

**Table 3:** Percentage corrosion inhibition efficiency and EIS parameters of varied concentrations of BLE extract for X52-steel in 1.0 M HCl at 298 K calculated by EIS method.

BLE (mg L^−1^)	*R*_p_ (ohm cm^2^)	CPE (Y_0_ × 10^6^ (Scm^−2^ S^n2^)	n	χ^2^	C_dl_ (µF cm^−2^)	*η*_R_%
Blank	53. 7	85.8	0.754	1.43 × 10^−3^	527.3	-
25	84.3	74.2	0.877	1.94 × 10^−3^	467.2	36.2
50	234.6	55.2	0.886	2.03 × 10^−3^	332.6	77.1
100	295.7	35.6	0.891	1.61 × 10^−3^	203.5	81.8
200	345.8	25.8	0.823	1.09 × 10^−3^	145.3	84.4
300	799.2	23.5	0.811	2.15 × 10^−3^	128.4	93.2
400	855.8	9.6	0.893	1.20 × 10^−3^	60.6	93.7

where the *R*_p(0)_ is the polarization resistance without BLE extract.

Table 3 displays that as the concentration of BLE extract rose, the values of *R*_p_ increased and *C*_dl_ decreased, implying that the production of a protective film on the X52-steel surface can lead to the creation of a more uniform protective layer to delay the transmission of corrosive species. Furthermore, when compared to the blank experiment, the value of CPE with various BLE extract concentrations drops dramatically, which is believed to be due to the BLE extract molecules going to replace the water molecules adsorbed on the X52-steel surface, indicating a thicker layer between X52-steel and solution^35^.

The equivalent circuit fitted is valid based on the obtained minimum goodness of fit (chi-square = χ^2^) value. Table 3 reveals that the values of *n* (surface heterogeneity) rise in comparison to the control solution, which can be attributed to the adsorbed BLE extract molecules' ability to form more homogeneous surface species.

In the presence of 400 mg L^−1^, the inhibition efficiency (*η*_R_%) achieved a maximum of 93.7%, indicating that the BLE extract has a clear inhibitory action owing to adsorption on the X52-steel surface.

### Adsorption isotherms

To establish relevant insights on the interaction of BLE extract and X52-steel surfaces, adsorption isotherms are used. Based on the adsorption concept, the replacement of BLE extract molecules BLE_(sol)_ for water molecules occurs during the adsorption of BLE extract on X52-steel surfaces^36^:

9 $${\text{BLE}}_{{({\text{sol}})}} + _{{\text{n}}} {\text{H}}_{{2}} {\text{O}}/{\text{X52}} - {\text{steel}} \leftrightarrow _{{\text{n}}} {\text{H}}_{{2}} {\text{O}}_{{({\text{sol}})}} + {\text{BLE}}/{\text{X52 - steel}}$$

As a result, the data collected from the weight loss approach (see Table 1) was used to simulate four isotherms: Langmuir, Freundlich, Temkin, and Frumkin. The Langmuir isotherm, which may be described by Eq. (10), is the result that best fits.

10 $$\frac{{C_{{{\text{inh}}}} }}{\theta } = \frac{1}{{K_{{{\text{ads}}}} }} + C_{{{\text{inh}}}}$$

where *C*_inh_ = BLE extract concentration, *K*_ads_ = constant equilibrium of adsorption.

**Figure 8:**
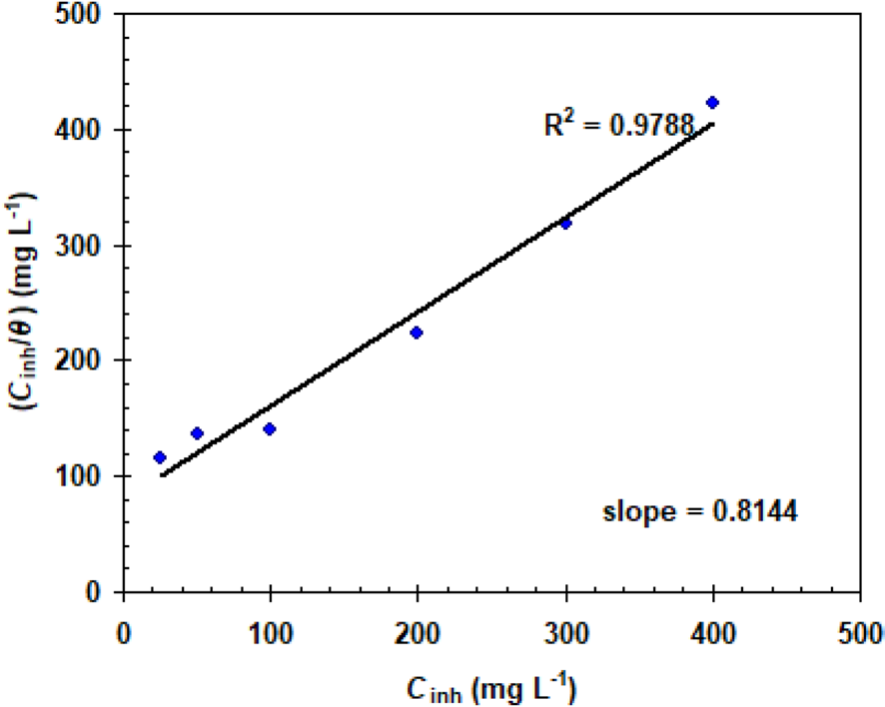
Langmuir isotherm for the studied BLE extract at 298 K.

The model using Langmuir isotherm is shown in Fig. 8. The adsorption activity follows the Langmuir adsorption theory, as indicated by the correlation coefficient (R^2^ > 0.97), establishing a protecting mono-layer of BLE extract molecules on the X52-steel surface.

Indeed, merely using R^2^ values to fit experimental data to the Langmuir adsorption is insufficient. This issue could be overcome by including a "dimensionless separation constant," (*K*_L_) (see Eq. 11)^37^.

11 $$K_{{\text{L}}} = {1}/({1} + K_{{{\text{ads}}}} C_{{{\text{inh}}}} )$$

The adsorption operation is regarded favourable since the values of *K*_L_ are less than one (see Table 4), and the experimental results satisfy the Langmuir adsorption isotherm.

**Table 4:** Dimensionless separation factor (*K*_L_) varied concentrations of BLE extract for X52-steel in 1.0 M HCl at 298 K.

BLE (mg L^−1^)	*K*_L_
25	0.783
50	0.617
100	0.446
200	0.287
300	0.211
400	0.187

The small *K*_ads_ value (0.0124 L/mg) validates BLE extract's physical adsorption activity^38^. Because the molecular mass of BLE extract molecules is uncertain, it is not feasible to assess the free standard adsorption energy, as stated by numerous other studies^39^.

### Surface morphologies

**Figure 9:**
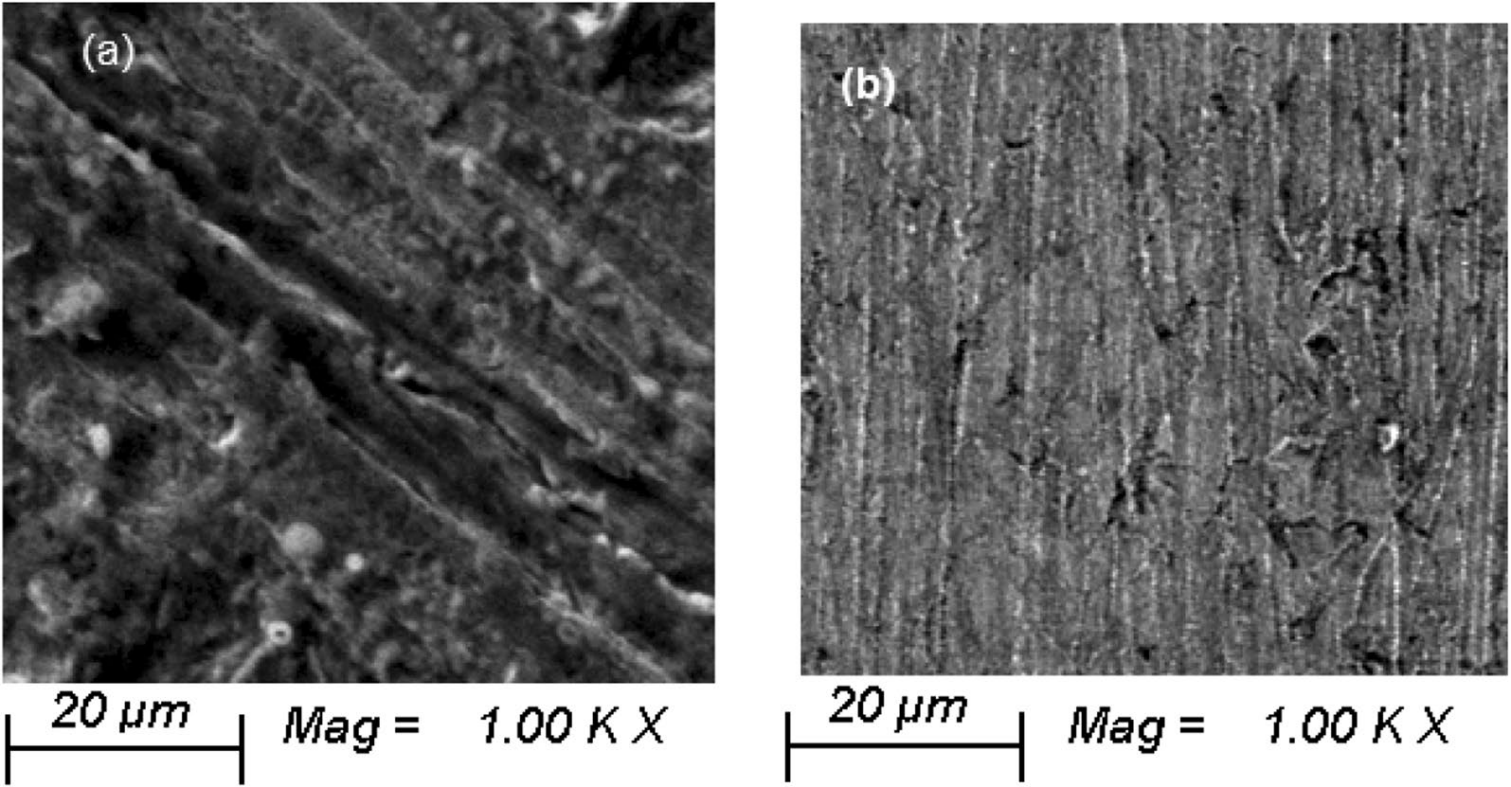
SEM images for X52-steel corrosion in pickling solution (1.0 M HCl) without (**a**) and with (**b**) 400 mg L^−1^ of BLE extract at 298 K.

SEM was used to examine the surface morphology of X52-steel surfaces that had been immersed in the pickling solution for 5.0 h without and with (400 mg L^−1^) BLE extract. The pickling solution significantly damages the surface of the X52-steel surface, as seen in Fig. 9a: pores form on the X52-steel surface plate, which is accompanied by surface roughness. SEM images of specimens dipped in BLE extract-treated BLE extract, on the other hand, reveal a better uniform and clean texture (Fig. 9b).

**Figure 10:**
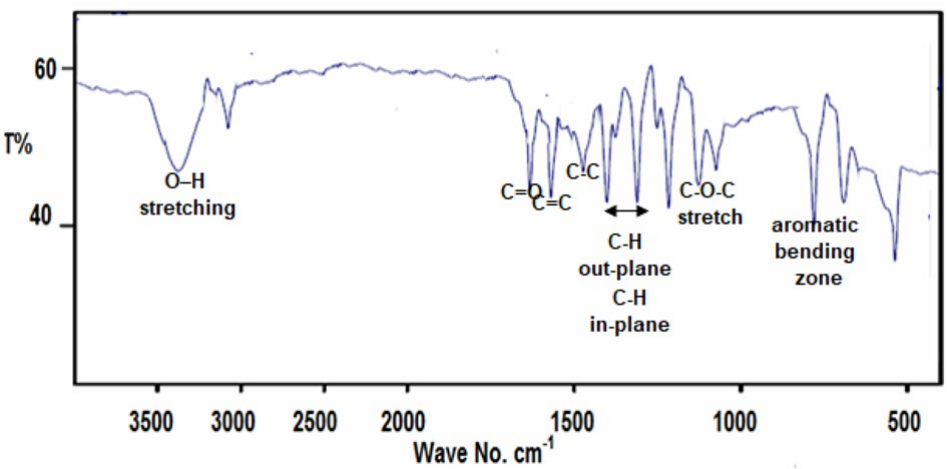
FT-IR spectra of the film formed on the surface of X52-steel in the inhibited solution (1.0 M HCl + 400 mg L^−1^ of BLE extract).

We analyzed the FT-IR spectra of the scratched layer generated on the X52-steel surface upon electrode soaking in pickling solution including 400 mg L^−1^ of BLE extract to reveal the adsorption procedure of BLE extract molecules on the X52-steel surface (Fig. 10). The adsorption of BLE extract molecules on the X52-steel surface is recognized by matching the FT-IR spectra in Figs. 3 and 10. The majority of the typical bands in Fig. 3 are found at comparable frequencies in Fig. 10, but with different signal strengths. This variance might be caused by the interaction of certain bioactive constituents of BLE extract molecules with the X52-steel, which results in the formation of a protective layer^40^.

### Inhibition mechanism

The various data (chemical and electrochemical techniques, SEM, FT-IR, and adsorption isotherms) demonstrate that the adsorption of bioactive chemical constituents in the BLE extract is the principal cause of corrosion protection of the X52-steel in the pickling solution. The HPLC examination identified several triterpenoids (betulin, betulinic acid, oleanolic), sitosterol, and kaempferol that are likely participating in the corrosion inhibition process. These molecules have O-bearing groups which possess a high binding affinity for Fe on the surface of X52-steel. Besides, the binding of aromatic rings, these molecules are attached through physisorption at the steel surface via partial exchange of O electrons and the creation of double bonds^41^. The organic compound-adsorbed layer acts as a shield between the metallic surface and the pickling solution^42,43^. The majority of the chemicals contained in BLE extract in 1.0 M HCl are neutral or cationic compounds. X52-steel, on the other hand, is expected to be positively chargeable in 1.0 M HCl. As a result, at 1.0 M HCl, the X52-steel surface will initially bind anionic ions that may cover the surface; however, in a second stage, the association of these anionic charges can assist in the adsorption of BLE cationic compounds. Because the organic molecules in BLE extract have greater dipole moments than water, they readily substitute water molecules at the surface of the X52-steel^44^. Table 5 compares the current study’s findings to previous work done by other researchers and demonstrates that the BLE extract can be highly efficient anti-corrosive agents at lower dosages^14,15,39,45,46^.

**Table 5:** Comparative studies have been done on extract green inhibitors for steel in acidic solutions.

Studied extract	Solutions	Extract concentrations	% Inhibition efficiency	Ref
Moluccensis *extract*	1 M HCl	500 mg L^−1^	68%	^45^
Terminalia arjuna leaves extract	0.2 M HCl	5 g extract/100 solution	64.1%	^46^
Olive leaves extract	2 M HCl,	900 mg L^−1^	93%	^14^
Henna extract	1 M HCl	1.2 g L^−1^	92.06%	^15^
Crude extract of a marine sponge	1 M HCl	2.0 g L^−1^	82%	^39^
BLE extract	1 M HCl	400 mg L^−1^	95.1%	This work

## Conclusions

In pickling solution, BLE extract indicates an effective inhibitor for corrosion of X52 pipeline steel. The presence of triterpenoids (betulin, betulinic acid, oleanolic), sitosterol, and kaempferol were identified by the chromatogram. The physisorption of the compounds included in the BLE extract is the cause of inhibition. These compounds protect the surface of X52-steel sheets, minimizing pickling solution corrosive damage. The EIS plots imply that the inhibitory effect improves with rising BLE dosage: the *R*_ct_ increases significantly while the CPE declines dramatically. Importantly, the polarization findings show that the BLE extract acts as a mixed inhibitor. The adsorption activity of BLE extract is consistent with the Langmuir adsorption theory, as evidenced by SEM and FTIR spectra. This research opens up a new avenue for environmentally corrosion inhibitors (i.e. BLE extract).for pipe steel that must be cleaned using acid chemicals.

## Data Availability

The datasets used and/or analysed during the current study available from the corresponding author on reasonable request.
