# Author Correction: Utilizing birch leaf extract in pickling liquid as a sustainable source of corrosion inhibitor for pipeline steel

**DOI:** 10.1038/s41598-023-27865-0

**Published:** 2023-01-12

**Authors:** Q. Mohsen, M. A. Deyab

**Affiliations:** 1 Department of Chemistry, College of Sciences, Taif University, Taif, Saudi Arabia; 2 Egyptian Petroleum Research Institute (EPRI), Nasr City, Cairo Egypt

Correction to: *Scientific Reports* 10.1038/s41598-022-23037-8, published online 11 November 2022

The original version of this Article contained an error in the Acknowledgements section. The name of the university was incorrect.

“Aif University Researchers Supporting Project number (TURSP—2020 /19), Taif University, Saudi Arabia.”

now reads:

“Taif University Researchers Supporting Project number (TURSP—2020 /19), Taif University, Saudi Arabia.”

The original Article has been corrected.

